# Supplementary material for: Complexome profiling of the Chlamydomonas psb28 mutant reveals TEF5 as an early PSII assembly factor
Source: Plant Cell. 2025 Mar 17;37(6):koaf055. doi: 10.1093/plcell/koaf055 (PMC12164590; doi:10.1093/plcell/koaf055)
Supplement: koaf055_Supplementary_Data [file koaf055_supplementary_data.zip › psb28_interactive.zip!/plots/intensity/qualityPlots/peptides/TRXO_Cre05.g248500.t1.2.html]

### Cre05.g248500.t1.2

Trivial name: TRXO  
SeqCoverage [median]: 52.00  
Mass: 16.01 kDa  
  
 

| Sequence | MissedCleavage | Mass [Da] | wtIntensity | muIntensity | Score |
| --- | --- | --- | --- | --- | --- |
| ADMLESLIQK | 0 | 1146.60 | 324.54 | 7732.22 | 128.28 |
| IDIDNTALGNTVNDHSITGVPTFVYYK | 0 | 2966.47 | 777.14 | 0.00 | 48.29 |
| MIAPVFSSLSNK | 0 | 1292.68 | 0.00 | 8145.75 | 101.97 |
| VVEVTSDQDFSAK | 0 | 1423.68 | 2301.16 | 6673.40 | 196.76 |
| YTDVTFVK | 0 | 971.50 | 0.00 | 5027.39 | 86.95 |
|  |  |  | 3402.8 | 27578.8 |  |
